# My Double Trouble: An Autobiographical Case Report of Psoriasis and Psoriatic Arthritis

**DOI:** 10.7759/cureus.20617

**Published:** 2021-12-22

**Authors:** Richard G Trohman

**Affiliations:** 1 Cardiac Electrophysiology, Rush University Medical Center, Chicago, USA

**Keywords:** autobiographical case report, biologics, systemic agents, phototherapy, topicals, cytokines, genetics, psoriatic arthritis, psoriasis

## Abstract

Psoriasis and psoriatic arthritis are overlapping, related, but distinct entities. Each occurs as a result of a complex combination of precipitants, genetic predispositions, and variable expression of a self-sustaining proinflammatory state. My case history and clinical course are outlined below. In addition, the epidemiology, pathogenesis, and the expanding armamentarium of treatment options, including their strengths and weaknesses, are discussed in detail.

## Introduction

Psoriasis and psoriatic arthritis are heritable autoimmune disorders. Although a great deal of progress has been made in understanding and treating these conditions, significant gaps remain in our understanding of the pathophysiology of these entities. Although treatment options have expanded, therapy is not without risk and the possibility of cure remains elusive.

According to the World Psoriasis Day consortium, 125 million people worldwide are afflicted with psoriasis. Approximately 1/3 of patients with psoriasis will develop psoriatic arthritis [1]. While arthritis may be painful and debilitating, involvement of the skin is associated with symptoms like itching or burning and very significant social stigmata. In one survey, 50% of participants noted they find patients with psoriasis unattractive. Responses from others range from staring to requests to leave public venues. Shame and embarrassment are common among psoriatic patients, and these feelings may have a profound effect on their quality of life [2]. I have coped with both disorders for many years, but I have not completely escaped their psychological impact.

My personal experience as well as a review of current knowledge and treatment options are included below.

## Case presentation

I have been afflicted with psoriasis since I was 14 or 15 years old. At first, it was only present on the right side of my forehead near the scalp line. A dermatologist misdiagnosed it as eczema and placed me on a topical steroid (which I used infrequently because it felt greasy). 

Like most young people, I was afraid of rejection. The plaque was readily visible, and I was concerned about its potential impact on my social life. The area affected did not change much for the next 10 years. As a fourth-year medical student rotating through dermatology, my attending physician told me that the plaque on my forehead was psoriasis. After reading and studying a bit, I accepted his diagnosis as correct.

**Figure 1:**
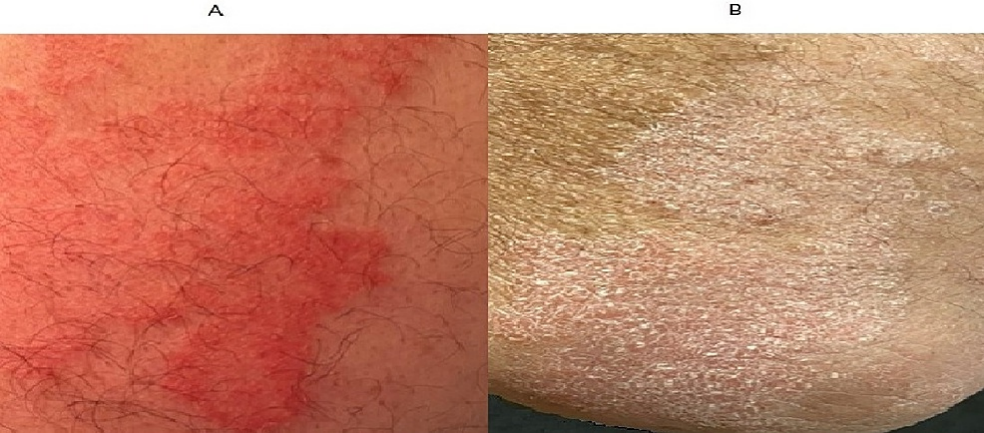
Psoriatic Lesions Diffuse psoriasis on the extensor surface of the arm. A) The upper arm is erythematous but not scaly. B) The elbow region appears pink, dry, and scaly.

Sometime during medical school and completion of my postgraduate training, my psoriasis worsened and typical psoriatic plaques became widespread (Figure 1). I also noted typical pitting of my nails and emerging evidence of dystrophy (thickening and discoloration, especially of my toenails). I found a new dermatologist who biopsied one peri-umbilical lesion. The result was something indefinite, along the lines of “possibly compatible with psoriasis."

The dermatologist recommended that I bathe less (hot showers and baths can dry your skin and cause psoriasis to flare) and prescribed another greasy topical steroid. I decided that smelling poorly was unlikely to enhance my doctor-patient interactions and that smearing my body with “grease” was not going to happen.

I married after finishing my training. Between 1983 and 1987, I lived and worked in South Florida. The warm, sunny climate did wonders for my psoriatic plaques. However, when I ran or jogged it frequently felt as though the femoral head had “popped” out of my left hip. I’d lift and rotate the leg and it felt better quickly. Typically, I tried to ignore it. 

At the age of 29, I moved to Pennsylvania for a new job. Shortly after moving in, while descending the stairs in my new home, I felt pain in my left lower back which radiated down my left leg. The pain persisted and was associated with intermittent paralysis of several toes. A myelogram was unremarkable (except for leaving me with a headache for several days). A CT scan suggested a herniated disc at L5. 

I underwent surgery which was supposed to last about 30 minutes. It lasted four hours! While in the recovery room, my surgeon came to speak to me. He asked me how I felt and I replied “OK”. He then told me that when he opened up my back, he uttered an expletive. My first thought was that I had cancer. Fortunately, he quickly explained that he had found extensive scar tissue and no evidence of a herniated disc. Removing the scar tissue was apparently quite complex.

My recovery was sufficient to resume work and even wear a lead apron during fluoroscopy. However, I couldn’t run or play basketball or softball. I walked and played some golf without significant discomfort. 

We moved to Cleveland for nearly eight years and then to Chicago. After moving to Chicago, I remained able to play golf (albeit, still poorly) without physical hindrance. Nevertheless, as I reached my early 40s, I noticed that my hands were swelling during golf and looked like sausage links. The first time it happened, I thought it was an allergic reaction to the surrounding flora. I quickly realized that it was a manifestation of psoriatic arthritis. A rheumatologist confirmed the diagnosis of psoriatic arthritis and recommended methotrexate (MTX), but I was hesitant about the risk (albeit very small) of retroperitoneal fibrosis. I managed my arthritic discomfort with ibuprofen or naproxen. Unfortunately, I became more sedentary and gained a considerable amount of detrimental weight.

I herniated a disc in my neck by tripping on the steps and falling out of my son’s music tour bus but recovered well after surgery. In a subsequent automobile accident, I fractured C2. Remarkably, the fractured segments ran vertically and did not impinge on my spinal cord. I recovered without surgical intervention.

After a particularly bad psoriatic outbreak, I decided to try to improve control of my psoriatic plaques. A brief trial of phototherapy with UVB light was not very helpful and the time commitment (see below) was a major challenge because of my clinical responsibilities. One of the dermatologists recommended steroid ointment and covering the affected areas with plastic wrap. Needless to say, I rejected this recommendation. A second dermatologist was willing to discuss and try treatment with Anti-TNF-α monoclonal antibodies. I injected adalimumab twice a month and had a splendid response for six months. Unfortunately, adalimumab stopped working. I hoped to try etanercept or infliximab, but my health insurance refused to pay for it.

My wife died of a glioblastoma in 2015. Although I was devastated, I trudged on. As I reached my late 50s, I started having radicular pain down my left leg. I responded to a single epidural steroid injection and did reasonably well for 12-18 months. A similar bout of radicular pain responded to two epidural steroid injections for about the same duration. Unfortunately, my third bout failed to respond to three epidural steroid injections. 

Ultimately, I returned to the rheumatologist noted above. He assured me that further investigation had determined that the risk of retroperitoneal fibrosis with MTX was 1/10th lower than he originally told me. I agreed to start this therapy and gradual dose titration relieved my back pain. My skin improved, although it has not been as clear as it was when adalimumab was working.

After 24 years in Chicago, I remarried, retired, and moved to Cincinnati, Ohio in 2020. Part-time clinical work is on my horizon. My new rheumatologist has discussed adding intravenous (biologic) therapy to my regimen. Although insurance funds it, and I am vaccinated, we agreed it is unwise to increase my immunosuppression during the COVID-19 pandemic.

Beyond the problems I have had with my back, psoriasis has not impeded me. Like everyone I have had my ups and downs, but psoriasis and psoriatic arthritis were issues I dealt with, not reasons to feel sorry for myself.

## Discussion

Epidemiology and pathogenesis

There are five types of psoriasis: plaque psoriasis (or psoriasis vulgaris), guttate or eruptive psoriasis, inverse psoriasis (also known as intertriginous or flexural psoriasis), as well as pustular psoriasis and erythrodermic psoriasis which are more serious forms of the disease. I have plaque psoriasis, which is the most common form and accounts for 80-90% of cases [3,4].

Psoriasis can begin at any age, but has two peaks of onset, first at age 20 to 30 years and second at age 50 to 60 years [5]. Common sites of skin involvement include the extensor surfaces of the forearms (Figure 1) and shins, peri-umbilical, perianal, retro-auricular regions, and scalp [3].

It is possible (but unlikely) that I had childhood psoriasis which can involve the face. Children with psoriasis often present with guttate (teardrop) lesions. About 1/3 of children with guttate psoriasis will develop plaque psoriasis later in life [3,6].

A genetic predisposition is the main risk for the development of psoriasis. More than 80 risk loci have been identified and account for 30% of disease heritability [6]. The major genetic risk factor for early-onset psoriasis is HLA (human leukocyte antigen)-C*06:02. Nevertheless, psoriatic arthritis is not associated with HLA-C*06:02 and this locus appears to actually decrease the likelihood of developing psoriatic arthritis [7,8]. HLA-B*08, HLA-B*27, HLA-B*38, and HLA-B*39 have been associated with the greatest risk of psoriatic arthritis [8]. It has been estimated that up to 30% of patients with psoriasis develop concomitant psoriatic arthritis [8]. Although psoriasis and psoriatic arthritis have overlapping features, they are believed to be distinct genetic, immunological, and therapeutically responsive entities. About 40-50% of people with psoriatic arthritis have the HLA-B*27 genotype [8].

I have not been able to uncover any evidence of psoriasis or psoriatic arthritis in my ancestry. My mother suffered from rheumatoid arthritis and my father had polymyalgia rheumatica. The HLA-DR4 and HLA-DR1 alleles have been associated with both of these entities [9,10]. The HLA locus is located on the short arm of chromosome 6 and covers a 7.6 Mb region with > 250 highly polymorphic genes. The high gene density, including clusters with related functions, numerous polymorphisms, and linkage disequilibrium (some alleles are grouped together more often than expected) has made it difficult to comprehensively unravel HLA functions [11]. It is tempting to speculate that my parents had HLA-related rheumatological disorders that are associated with mine, but conclusive evidence is definitely lacking.

Psoriasis does not manifest unless an environmental trigger, such as stress, infection (particularly streptococcal), alcohol consumption, smoking, exposure to drugs such as lithium, antimalarials, and non-steroidal inflammatory agents, and, in some cases, sunlight results in a gene-environment interaction [7]. I recall experiencing significant stress due to parental discord several years before my psoriasis outbreak. However, about three to four uneventful years elapsed between the stress and the appearance of the plaque on my forehead. I did not drink alcohol or take any of the aforementioned pharmaceuticals. As noted above, sunlight improved my psoriasis. To the best of my knowledge, I have not had a streptococcal infection. Nevertheless, upper respiratory infection (URI) of viral origin has been implicated in acute psoriasis flares [12]. I had a fair number of URIs as an adolescent, perhaps one was the trigger.

T cells play an important, but not exclusive, role in the pathogenesis of psoriasis. Neutrophils, dendritic cells, and keratinocytes also play an important role in the aberrant immune response [7].

Communication between these cells occurs primarily through cytokines such as Tumor Necrosis Factor-alpha (TNF-α), interleukins (IL-17, IL-22, IL-23, IL-36 and through activation of keratinocytes (which regulate IL-36), driving epidermal hyperproliferation, as well as production of antimicrobial proteins, growth factors, and chemokines. These cytokines promote changes characteristic of psoriasis including angiogenesis, neutrophil infiltration, and increased numbers of helper T (Th 1and Th 17) cells. Aberrant activation of tissue-resident memory T cells (TRM), which persist long-term in the skin, by autoantigens contributes to immune-mediated diseases. Pathogenic autoreactive TRM contributes to the induction of psoriatic skin lesions [7,13].

Cytokines facilitate interactions between key cells and the balance between different cytokines may account for variations in disease presentation [7]. Th 1’s main cytokine is interferon-gamma (IFN-γ) and Th 17 cells produce IL-17. IL-17 and IL-23 dominate in plaque psoriasis and are amplified by IFN-γ and TNF-α. IL-17, in turn, promotes IL-36 expression and activation [7]. These cells and cytokines promote a self-sustaining pro-inflammatory state [7,14-15].

Regulatory T cells (Treg) are a controversial entity. It has been proposed that they suppress potentially deleterious activities of Th cells [16].

Treatment strategies

Although psoriasis and psoriatic arthritis share some pathogenetic and immunological features and their therapies frequently overlap, they are distinct genetic, immunological, and therapeutically responsive entities [7]. As I have noted, there are a variety of treatment options for psoriasis and psoriatic arthritis. Their strengths and weaknesses are outlined below.

Although definitions vary, moderate-to-severe psoriasis may be accompanied by depression or anxiety and associated with functional impairment or significant distress [7]. Lifestyle modifications include addressing potential triggers via smoking cessation, reducing alcohol intake, losing weight, improving sleep, and exercise. I have taken off about 100 lbs. and exercise regularly. Although these measures have improved my overall well-being, they have not done much for my skin or arthritis.

Emollients (moisturizers) hydrate, soften, protect and lubricate the skin and make it look better. Topical therapies including corticosteroids, vitamin D3 analogs, calcineurin inhibitors, keratolytic, and combination agents are available in ointment, cream, foam, or gel preparations. Poor adherence to this treatment modality is a major limitation [7]. Based on my experience, this is not surprising.

Narrowband ultraviolet B (NB-UVB) radiation is the most commonly used form of phototherapy and is typically delivered two to three times/week for 12-16 weeks. Each treatment lasts about 10 minutes, but it is estimated that each visit will last 30 minutes [7,17]. It is effective in depleting skin-infiltrating T cells from the epidermis and dermis of psoriatic plaques by inducing apoptosis. It also induces antigen-specific immunosuppression. The proposed mechanism for the efficacy of NB-UVB is the normalization of an imbalance of Th17 cells and functional Treg cells [18].

In addition to being time-consuming, phototherapy can cause sunburn (usually mild). Wrinkling and skin discoloration, similar to what occurs with cigarette smoking or aging may also occur [15]. There is some data that suggests NB-UVB does not increase the risk of skin cancer, but a cautious interpretation has been recommended [19].

Oral systemic agents were the staple treatment for moderate-to-severe plaque psoriasis before the advent of biologics. Commonly employed oral agents include methotrexate, cyclosporin, acitretin, fumarates, and apremilast [7].

Methotrexate (MTX) has been prescribed for psoriasis and psoriatic arthritis for more than 50 years. The usual maintenance dose is 15-20 mg taken once a week (I take 20 mg/week) [7]. Unfortunately, MTX bioavailability is reduced by 30% starting at 15 mg/week due to limitations in gut absorption. Splitting oral doses (two half-doses at eight-hour intervals on one day of the week) has been reported to improve bioavailability, tolerance, adherence, and efficacy [20]. Other regimens include divided dosing split between different days of the week [21]. Methotrexate is also available for subcutaneous injection. This route of MTX delivery bypasses first-pass metabolism in the gastrointestinal tract and may be an effective alternative in patients who have had an inadequate response or are intolerant of the oral formulation [20].

Methotrexate’s mechanism of action is most likely via 5-aminoimidazole-4-carboxamide ribonucleotide transformylase activity and increased adenosine production, causing inhibition of lymphocyte function [7]. Methotrexate’s adverse effects include bone marrow suppression (the most common cause of iatrogenic fatality), cirrhosis, nausea, vomiting, alopecia, teratogenicity (MTX is contraindicated during pregnancy), and pulmonary toxicity. Folic acid administration decreases the gastrointestinal and hematopoietic adverse effects caused by MTX [7].

MTX is now considered a “possible” inductive factor for retroperitoneal fibrosis (RPF). Reports of MTX causing RPF are extremely infrequent making the description of the pathogenesis elusive [22]. Paradoxically, there is (limited) data suggesting that methotrexate may be effective in combination with prednisone for the treatment of relapsing idiopathic retroperitoneal fibrosis [23].

Cyclosporin, a systemic calcineurin inhibitor, is used for short-term treatment (≤ 1 year due to risk of irreversible nephrotoxicity) of patients with severe plaque psoriasis or who are in crisis, and as a bridge therapy to long-term therapies such as biologics or other oral medications. Cyclosporine is highly likely to clear psoriasis rapidly and thoroughly [7,24]. Cyclosporin’s additional adverse effects include hypertension, increased susceptibility to infection, nausea, hirsutism, gingival hyperplasia, drug-drug interactions, and electrolyte disturbances.

Acitretin, a systemic synthetic retinoid (vitamin-A derivative), normalizes the proliferation of keratinocytes and exerts immunomodulatory effects that decrease proinflammatory cytokines, such as IL-6 and IFN-γ. It is used to treat severe psoriasis [7]. To avoid fetal harm, women who are pregnant or plan to become pregnant within the next three years should not use this agent. It is recommended that women of child-bearing age who plan to use acitretin use multiple forms of contraception. In addition, patients and clinicians need to be aware that acitretin interferes with the action of microdose progestin oral contraceptives. The combination of alcohol ingestion and acitretin may also result in fetal harm [25].

Fumaric acid esters and dimethyl fumarate are used to treat moderate to severe psoriasis. They inhibit the maturation of dendritic cells, induce T cell apoptosis, and interfere with leukocyte extravasation. Nausea and vomiting affect up to 40% of recipients. Lymphocytopenia ≤ 0.7 K/μL should trigger dose reduction because of the risk of progressive multifocal leukoencephalopathy [7].

The phosphodiesterase-4 inhibitor apremilast decreases proinflammatory cytokines (such as TNFα, IL-2, and IL-12) and increases anti-inflammatory cytokines (such as IL-10). It is used to treat moderate to severe psoriasis. Gastrointestinal side effects include diarrhea, nausea, and weight loss [7].

During the past two decades, the development of biologics has revolutionized the treatment of psoriasis and psoriatic arthritis. In psoriasis and psoriatic arthritis, excess production of TNF-α leads to the rapid growth of skin and/or damage to joint tissue. TNF-α blockers (listed first by generic then brand name) include certolizumab pegol (Cimzia), etanercept (Enbrel), adalimumab (Humira), golimumab (Simponi and Simponi Aria), and infliximab (Remicade)26. Golimumab is currently approved for the treatment of psoriatic arthritis but not psoriasis [7].

Ustekinumab (Stelara) blocks the common p40 subunit of interleukin 12 (IL-12) and interleukin 23 (IL-23) which is associated with psoriatic inflammation. Three additional agents Guselkumab (Tremfya), risankizumab-rzaa (Skyrizi), and tildrakizumab-asmn (Ilumya) target the p19 subunit of IL-23 [7,26].

IL-17 inhibitors secukinumab (Cosentyx) and ixekizumab (Taltz) specifically target IL-17A and brodalumab (Siliq) targets the IL-17 receptor A unit (IL-17RA), inhibiting IL-17A, IL-17F, and two other members of the IL-17 cytokine family (IL-17C and IL-17E or IL-25) [7,26]. 

Abatacept (Orencia) was approved in 2017 for the treatment of psoriatic arthritis. This agent reduces inflammation by inhibiting T-cells from becoming activated [26].

Patients’ responses to biologics vary. Some individuals do not respond at all. Much more commonly, an initial response is lost after months to years [7]. Although secondary treatment failure is less common in men, that was the experience I had with adalimumab (Humira). Some patients treated with anti-TNF-α and agents that target IL-6 will experience paradoxical worsening of their disease. This is more common in women and the exacerbation often presents as palmoplantar pustulosis [7].

After taking a biologic it is possible to experience allergic reactions ranging from mild redness, itchiness, and/or warm and tender skin around the injection site, or a full-body rash to anaphylactic shock. All biologics suppress the immune system and increase the risk of infections such as upper respiratory infections, pneumonia, urinary tract infections, and skin infections. Opportunistic infections like hepatitis B, tuberculosis, and fungal infections are also possible [27]. Available safety data has been reassuring suggesting no major risk for infection or cancer if pretreatment and annual screening are performed. The impact of psoriasis and its therapy on COVID-19 susceptibility and severity is uncertain [7]. 

Other side effects of biologics may include nausea, vomiting, diarrhea or constipation, cough, and feeling weak. Less commonly, patients may develop visual disturbances, swelling of the ankles and/or hands, numbness or tingling, joint pain, and rashes that are exacerbated by sun exposure [27].

Biosimilars are modeled after FDA-approved biologics. They are less expensive and provide cost savings in high-income countries. Their development may allow patients in low-income and medium-income countries to have greater opportunities to be treated [7,26]. A detailed discussion of the various biosimilars is beyond the scope of this manuscript.

I have attributed my back issues and surgical findings to psoriatic arthritis. Despite the somewhat unusual surgical findings, the evidence supports my contention. My arthritic problems began in my early 30s which is consistent with the psoriatic arthritis age onset (30-50 years old) [28]. Back pain often begins before age 40.

Sacroiliac joint involvement may be one of the earliest manifestations of psoriatic arthritis. Some authors believe that radiologic evidence of psoriatic spinal disease may precede clinical symptoms [29]. There is evidence that nerve entrapment from fibrosis can cause sciatica [30]. The sustained relief that MTX provides suggests that my pain is related to inflammation.

On the other hand, there is conflicting evidence on whether inflammatory involvement of large peripheral nerves with psoriasis or psoriatic arthritis is likely. One study suggested a high prevalence of neuropathic-like pain in psoriatic arthritis [31]. Another study employed nerve conduction velocity assessment and found no measurable abnormalities of the peripheral large nerve fibers in psoriatic patients and concluded an association of psoriasis with peripheral large fiber neuropathy cannot be suggested [32]. A case report describes psoriatic skin lesions in the distribution of the sciatic nerve, but there was concomitant lumbar disk herniation [33]. Evidence in favor of a neurocutaneous pathway in psoriasis has been provided by Zhu et al.[34] who described patients that experienced unilateral improvement and or complete remission of plaque psoriasis following nerve damage in the affected dermatomal region.

Psychological and socioeconomic impact

The impact of psoriasis on patients extends well beyond the physical manifestations. It can have a profound psychosocial effect that does not necessarily correlate with skin lesion severity [35-36], The presence of psoriatic plaques can result in negative reactions from other people, including repulsion and fear [35].

In the aforementioned, survey [2] 61% of lay observers believed psoriatic lesions were contagious because of their scale, color, and size. Psoriasis can affect a patient's interpersonal relationships and limit intimacy. Among the same individuals surveyed, 21.4% said they would divorce or separate from a significant other because of their psoriasis [2]. 

Therefore, psoriatic stigmata may result in anxiety, depression, and social isolation. The psychosocial aspects of the disease may lead to patients believing their condition is more severe than their physician’s assessment [35]. Depression is more prevalent in psoriasis patients compared to the general population and they are more likely to have suicidal thoughts or to attempt suicide [35-37].

About half of patients are dissatisfied with their treatment and 40% do not use their therapy as prescribed [35]. Although it has been suggested that those who receive biologics have higher treatment satisfaction, the 2014 Multinational Assessment of Psoriasis and Psoriatic Arthritis Survey reported that among patients who had received oral or biologic therapy, 57% and 45%, respectively, discontinued therapy, most often for safety/tolerability reasons and a lack/loss of efficacy [38]. 

Compounding these issues, moderate-to-severe psoriasis has a negative impact on employment opportunities and career prospects. The level of income in psoriasis patients is inversely correlated with disease severity [35]. Concomitant symptoms of psoriatic arthritis such as fatigue, joint pain and stiffness, back pain (with or without sciatica), enthesitis (inflammation and pain where ligaments and tendons attach to bone) have been associated with rates of absenteeism from work and diminished productivity at work [8]. Less commonly, dyspnea (from psoriatic lung involvement) or cardiovascular events may make working impossible [8,39]. Financial hardship and debt are associated with anxiety and depression. This may also culminate in suicidal ideation, suicide attempt(s), or suicide completion. There is evidence that physical health is also negatively impacted by financial woes [40-41]. Lack of insurance or optimal coverage (which is often related to socioeconomic status) may impact treatment availability [35]. As noted above, biosimilars may help relieve some of the cost issues associated with biologics, however, they are by no means a panacea.

The unprecedented societal shock triggered by the COVID-19 pandemic has led to a higher prevalence of adverse mental health, including feelings of depression and anxiety about physical health. Feelings of loneliness and isolation have been exacerbated by government imposed lockdowns. Economic hardship during the pandemic has been less among individuals with greater “occupational prestige” (earnings and skill level) [42]. Involuntary reduction in work hours, loss of income, and/or employment has been concentrated among those with lower prestige-ranked occupations. Likewise, individuals with lower incomes have been more likely to suffer depression [42]. Given the known economic hardships that face many psoriasis sufferers, there is little doubt that their shame and fear are exacerbated by the possibility of looming job loss [42]. These issues have made obtaining and affording appropriate medical therapy even more difficult.

## Conclusions

Psoriasis and psoriatic arthritis are heritable, auto-immune disorders. Although the main risk for the development of psoriasis is a genetic predisposition, I do not have any family members with either entity. It is clear that T cells, neutrophils, dendritic cells, keratinocytes, and cytokines play a significant role in the pathogenesis of these entities. Communication between these cells, primarily through cytokines, plays a key role in creating an aberrant self-sustaining pro-inflammatory state. Gene-environment interaction is pivotal to the manifestation of these disorders. Lifestyle modifications may limit the extent of disease and prevent flare-ups. About 1/3 of patients with psoriasis develop psoriatic arthritis, usually years after the dermatological manifestations appear. The spine is commonly involved and maybe an early manifestation of the arthritic entity.

Older therapeutic options such as the application of topicals, phototherapy, and oral-systemic agents remain in use today. Progress in the last 20 years has led to the development of biologicals that are more efficacious. All patients do not respond in the same manner to biologicals. Secondary failure may occur after initial success. Although combination therapy may be safely prescribed, it is crucial to remember that pharmacological therapies may have serious adverse effects. Management of psoriasis and psoriatic arthritis remains challenging. People who suffer from psoriasis are often anxious and depressed. The response of others to skin lesions is frequently negative and contributes significantly to psychological impairment. I have been lucky to have avoided frank disdain, but remain a bit insecure about my appearance. For many less fortunate psoriasis sufferers, the concept that beauty is only skin deep is, at best, far from a reality.
